# Endpoint regularity of discrete multisublinear fractional maximal operators associated with $\ell^{1}$-balls

**DOI:** 10.1186/s13660-018-1627-9

**Published:** 2018-02-06

**Authors:** Feng Liu

**Affiliations:** College of Mathematics and Systems Science, Shandong University of Science and Technology, Qingdao, China

**Keywords:** 42B25, 46E35, 26A45, 39A12, Discrete multisublinear fractional maximal function, $\ell^{1}$-balls, Sobolev space, Bounded variation, Continuity

## Abstract

In this paper we investigate the endpoint regularity of the discrete *m*-sublinear fractional maximal operator associated with $\ell^{1}$-balls, both in the centered and uncentered versions. We show that these operators map $\ell^{1}(\mathbb{Z}^{d})\times\cdots\times \ell^{1}(\mathbb{Z}^{d})$ into $\operatorname{BV}(\mathbb{Z}^{d})$ boundedly and continuously. Here $\operatorname{BV}(\mathbb{Z}^{d})$ represents the set of functions of bounded variation defined on $\mathbb{Z}^{d}$.

## Introduction

### Background

The regularity theory of maximal operators has been the subject of many recent articles in harmonic analysis. The first work was contributed by Kinnunen [1] who investigated the Sobolev regularity of the centered Hardy–Littlewood maximal function $\mathcal{M}$ and proved that $\mathcal{M}$ is bounded on the first order Sobolev spaces $W^{1,p}(\mathbb{R}^{d})$ for all $1< p\leq\infty$. It was noticed that the $W^{1,p}$-bound for the uncentered maximal operator $\widetilde{\mathcal{M}}$ also holds by a simple modification of Kinnunen’s arguments or [2, Theorem 1]. Subsequently, the above result was extended to a local version in [3], to a fractional version in [4], to a multisublinear version in [5, 6] and to a one-sided version in [7]. Due to the lack of sublinearity of weak derivatives of the maximal function, the continuity of $\mathcal{M}: W^{1,p}\rightarrow W^{1,p}$ for $1< p<\infty$ is a certainly non-trivial problem. This question was resolved by Luiro in [8]. Later on, Luiro’s result was extended to the local version in [9] and to the multisublinear version in [5, 10]. Another way to extend the regularity theory of maximal operators is to study its behavior on different differentiable function spaces, such as fractional Sobolev spaces, Triebel–Lizorkin spaces, Besov spaces and so on. We refer the readers to consult [9, 11–13]. We notice that the $L^{p}$-bounds for $\mathcal{M}$ is the crux of the $W^{1,p}$-bounds for $\mathcal{M}$ for all $1< p\leq\infty$. Due to the lack of the $L^{1}$-bounds for $\mathcal{M}$, the $W^{1,1}$-regularity of maximal operators seems to be a deeper issue. A crucial question was posed by Hajłasz and Onninen in [2]:

#### Question A

([2])

Is the operator $f\mapsto|\nabla\mathcal{M}f|$ bounded from $W^{1,1} (\mathbb{R}^{d})$ to $L^{1}(\mathbb{R}^{d})$?

This question was solved completely in dimension $d=1$. Tanaka [14] first proved that $\widetilde{\mathcal{M}}f$ is weakly differentiable and satisfies

1.1 $$ \bigl\Vert (\widetilde{\mathcal{M}}f)' \bigr\Vert _{L^{1}(\mathbb {R})}\leq2 \bigl\Vert f' \bigr\Vert _{L^{1}(\mathbb{R})} $$

if $f\in W^{1,1}(\mathbb{R})$. The above result was later refined by Aldaz and Pérez Lázaro [15] who showed that if *f* is of bounded variation on $\mathbb{R}$, then $\widetilde{\mathcal{M}}f$ is absolutely continuous and

1.2 $$ \operatorname{Var} (\widetilde{\mathcal{M}}f )\leq\operatorname{Var}(f), $$

where $\operatorname{Var}(f)$ denotes the total variation of *f*. The above result directly yields (1.1) with constant $C=1$ (see also [16]). For the centered version, Kurka [17] showed that if *f* is of bounded variation on $\mathbb{R}$, then (1.2) holds for $\mathcal{M}$ with constant $C=240\mbox{,}004$. Kurka also observed that if $f\in W^{1,1}(\mathbb{R})$, then $\mathcal{M}f$ is weakly differentiable and (1.1) also holds for $\mathcal{M}$ with constant $C=240\mbox{,}004$. Recently, inequalities (1.1) and (1.2) were extended to a fractional setting in [18] and to a multisublinear setting in [19]. In the remarkable work [20], Carneiro et al. proved that the operator $f\mapsto(\widetilde{\mathcal{M}}f)'$ is continuous from $W^{1,1}(\mathbb{R})$ to $L^{1}(\mathbb{R})$. It is currently unknown whether the above continuity also holds for the centered version. For the general case $d\geq2$, Question A remains open, and partial progress was obtained by Hajłasz and Malý [21], Luiro [22] and Saari [23]. Other works on the endpoint regularity of maximal operators include [7, 24, 25].

### Discrete setting

We shall generally denote by $\vec{n}=(n_{1}, n_{2},\ldots,n_{d})$ a vector in $\mathbb{Z}^{d}$. For a discrete function $f:\mathbb{Z}^{d}\rightarrow\mathbb{R}$, we define the $\ell^{p}(\mathbb{Z}^{d})$-norm for $1\leq p<\infty$ by $\Vert f \Vert_{\ell^{p}(\mathbb{Z}^{d})}=(\sum_{\vec{n}\in\mathbb{Z}^{d}} |f(\vec{n})|^{p})^{1/p}$ and the $\ell^{\infty}(\mathbb{Z}^{d})$-norm by $\Vert f \Vert_{\ell^{\infty}(\mathbb{Z}^{d})}=\sup_{\vec{n}\in \mathbb{Z}^{d}}|f(\vec{n})|$. We also let $\Vert\vec{n} \Vert_{p}= (\sum_{i=1}^{d}|n_{i}|^{p})^{1/p}$ for all $1\leq p<\infty$. Formally, we define the discrete analogue of the Sobolev spaces by

$$W^{1,p}\bigl(\mathbb{Z}^{d}\bigr):=\bigl\{ f: \mathbb{Z}^{d}\rightarrow\mathbb{R}\mid \Vert f \Vert _{1,p}= \Vert f \Vert _{\ell ^{p}(\mathbb{Z}^{d})}+ \Vert \nabla f \Vert _{\ell^{p}(\mathbb {Z}^{d})}< \infty\bigr\} , $$

where ∇*f* is the gradient of a discrete function *f* defined by $\nabla f(\vec{n})=(D_{1} f(\vec{n}), \ldots,D_{d}f(\vec{n}))$ and $D_{l} f(\vec{n})$ is the partial derivative of *f* denoted by

$$D_{l} f(\vec{n})=f(\vec{n}+\vec{e}_{l})-f(\vec{n}) $$

and $\vec{e}_{l}=(0,\ldots,0,1,0,\ldots,0)$ is the canonical *l*th base vector, $l=1,2,\ldots,d$. It is clear that

1.3 $$ \Vert f \Vert _{\ell^{p}(\mathbb{Z}^{d})}\leq \Vert f \Vert _{1,p}\leq(2d+1) \Vert f \Vert _{\ell ^{p}(\mathbb{Z}^{d})}\quad\forall1\leq p\leq\infty, $$

which yields that the discrete Sobolev space $W^{1,p} (\mathbb{Z}^{d})$ is just $\ell^{p}(\mathbb{Z}^{d})$ with an equivalent norm. We also denote by $\operatorname{BV}(\mathbb{Z}^{d})$ the set of all functions of bounded variation defined on $\mathbb{Z}^{d}$, where the total variation of $f:\mathbb{Z}^{d}\rightarrow\mathbb{R}$ is defined by

$$\operatorname{Var}(f)=\sum_{l=1}^{d} \Vert D_{l}f \Vert _{\ell ^{1}(\mathbb{Z}^{d})}. $$

It follows that

1.4 $$ \Vert \nabla f \Vert _{\ell^{1}(\mathbb{Z}^{d})}\leq\operatorname{Var}(f)\leq d \Vert \nabla f \Vert _{\ell^{1}(\mathbb {Z}^{d})}. $$

Recently, the study of the regularity properties of discrete maximal operators has also attracted many scholars. This progress began with Bober et al. [26] who proved that

1.5 $$ \operatorname{Var}(\widetilde{M}f)\leq\operatorname{Var}(f) $$

and

1.6 $$ \operatorname{Var}(Mf)\leq\biggl(2+\frac{146}{315}\biggr) \Vert f \Vert _{\ell^{1}(\mathbb{Z})}, $$

where *M* (resp., *M̃*) is the usual discrete centered (resp., uncentered) Hardy–Littlewood maximal operator. We notice that inequality (1.5) is sharp. Subsequently, Temur [27] proved (1.5) for *M* (with constant $C=294\mbox{,}912\mbox{,}004$) following Kurka’s breakthrough [17]. Inequality (1.6) is not optimal, and it was asked in [26] whether the sharp constant for inequality (1.6) is in fact $C=2$. This question was resolved in the affirmative by Madrid in [28]. Recently, Carneiro and Madrid [18] extended inequality (1.5) to the fractional setting (also see [20, 29–31] for the relevant results).

For general dimension $d\geq1$, Carneiro and Hughes [32] studied the endpoint regularity of the discrete centered Hardy–Littlewood maximal operator associated with $\ell^{2}$-balls

$$Mf(\vec{n})=\sup_{r>0}\frac{1}{N(B_{r}(\vec{n}))} \sum _{\vec{k}\in B_{r}(\vec{n})\cap\mathbb{Z}^{d}} \bigl\vert f(\vec {k}) \bigr\vert , $$

where $B_{r}(\vec{n})$ is the open ball in $\mathbb{R}^{d}$ centered at *n⃗* with radius *r* and $N(B_{r}(\vec{n}))$ is the number of the lattice points in the set $B_{r}(\vec{n})$. Carneiro and Hughes [32] first proved that *M* and its uncentered version map $\ell^{1}(\mathbb{Z}^{d})$ into $\operatorname{BV} (\mathbb{Z}^{d})$ boundedly and continuously. The above result was later extended to a fractional setting in [18] and to a multisublinear setting in [33]. In particular, Liu and Wu [33] investigated the regularity of the discrete centered multisublinear fractional maximal operator associated with $\ell^{2}$-balls

$$\mathfrak{M}_{\alpha}(\vec{f}) (\vec{n})=\sup_{r>0} \prod_{j=1}^{m}\frac{1}{N(B_{r}(\vec{n}))^{1-\frac{\alpha}{md}}}\sum _{\vec{k}\in B_{r}(\vec{n})\cap\mathbb{Z}^{d}} \bigl\vert f_{j}(\vec {k}) \bigr\vert , $$

where $m\geq1$ and $0\leq\alpha< md$. Precisely, they proved the following result.

#### Theorem B

([33])

*Let* $d\geq1$ *and* $0\leq\alpha<(m-1)d+1$. *Then* $\mathfrak{M}_{\alpha}$ *maps* $\ell^{1}(\mathbb{Z}^{d})\times\cdots\times\ell^{1}(\mathbb{Z}^{d})$ *into* $\operatorname{BV}(\mathbb{Z}^{d})$ *boundedly and continuously*.

### Main results

It is well known that the geometry of $\ell^{1}$-balls in $\mathbb{Z}^{d}$ is more intricate than that of $\ell^{2}$-balls. Especially, the number of lattice points in the $\ell^{1}$-ball is more complex than that of $\ell^{2}$-ball. This makes the investigation of the discrete multisublinear maximal operators associated with $\ell^{1}$-balls very complex and interesting. The primary purpose of this paper is to explore the regularity properties of the discrete multisublinear maximal operators associated with $\ell^{1}$-balls. Let $m\geq1$ and $\vec{f}=(f_{1},\ldots,f_{m})$ with each $f_{j}$ being a discrete function on $\mathbb{Z}^{d}$. For $0\leq\alpha< md$, we define the discrete centered *m*-sublinear fractional maximal operator $\mathbf{M}_{\alpha}$ associated with $\ell^{1}$-balls by

$$\mathbf{M}_{\alpha}(\vec{f}) (\vec{n})=\sup_{r>0} \prod _{j=1}^{m}\frac{1}{N(\Gamma_{r}(\vec{n}))^{1-\frac{\alpha }{md}}}\sum _{\vec{k}\in\Gamma_{r}(\vec{n})} \bigl\vert f_{j}(\vec {k}) \bigr\vert , $$

where $\Gamma_{r}(\vec{n})$ is the $\ell^{1}$-ball centered at *n⃗* with radius *r*, i.e. $\Gamma_{r}(\vec{n}) =\{\vec{k}\in\mathbb{Z}^{d}; \Vert\vec{k}-\vec{n} \Vert_{1}< r\}$, and $N(\Gamma_{r}(\vec{n}))$ denotes the number of elements in the set $\Gamma_{r}(\vec{n})$. The uncentered version of $\mathbf{M}_{\alpha}$ is given by

$$\widetilde{\mathbf{M}}_{\alpha}(\vec{f}) (\vec{n})=\sup _{r>0,\vec {l}\in\mathbb{Z}^{d}\atop \vec{n}\in\Gamma_{r}(\vec{l})} \prod_{j=1}^{m} \frac{1}{N(\Gamma_{r}(\vec{l}))^{1-\frac{\alpha }{md}}}\sum_{\vec{k}\in\Gamma_{r}(\vec{l})} \bigl\vert f_{j}(\vec {k}) \bigr\vert . $$

Clearly, when $\alpha=0$ and $m=d=1$, $\mathbf{M}_{\alpha}=M$ and $\widetilde{\mathbf{M}}_{\alpha}=\widetilde{M}$. For the bounds of $\mathbf{M}_{\alpha}$ and $\widetilde{\mathbf{M}}_{\alpha}$, we have

1.7 $$ \bigl\Vert \widetilde{\mathbf{M}}_{\alpha}(\vec{f}) \bigr\Vert _{\ell^{q}(\mathbb{Z}^{d})}+ \bigl\Vert \mathbf{M}_{\alpha}(\vec{f}) \bigr\Vert _{\ell^{q}(\mathbb{Z}^{d})} \lesssim_{\alpha,m,d}\prod_{j=1}^{m} \Vert f_{j} \Vert _{\ell^{p_{j}}(\mathbb{Z}^{d})} $$

if $1< p_{1},\ldots,p_{m}\leq\infty$, $1\leq q\leq\infty$ for $\alpha=0$, and $1< p_{1},\ldots,p_{m}<\infty$, $1\leq q<\infty$ for $0<\alpha<md$, and $\frac{1}{q}\leq\frac{1}{p_{1}}+ \cdots+\frac{1}{p_{m}}-\frac{\alpha}{d}$. To see (1.7), we notice that

1.8 $$\begin{aligned}& B_{\frac{r}{\sqrt{d}}}(\vec{n})\cap\mathbb{Z}^{d}\subset\Gamma _{r}(\vec{n})\subset B_{r}(\vec{n})\cap \mathbb{Z}^{d}, \end{aligned}$$

1.9 $$\begin{aligned}& N\bigl(B_{\frac{r}{\sqrt{d}}}(\vec{n})\bigr)\leq N\bigl(\Gamma_{r}( \vec{n})\bigr)\leq N\bigl(B_{r}(\vec{n})\bigr). \end{aligned}$$

Here $B_{r}(\vec{n})$ is the open ball in $\mathbb{R}^{d}$ centered at *n⃗* with radius *r* and $N(B_{r}(\vec{n}))$ is the number of lattice points in the set $B_{r}(\vec{n})$. On the other hand, it was shown in [34] that

1.10 $$ c_{d}\biggl(r-\frac{\sqrt{d}}{2}\biggr)^{d}\leq N \bigl(B_{r}(\vec{0})\bigr)\leq c_{d}\biggl(r+ \frac {\sqrt{d}}{2}\biggr)^{d}\quad\forall r>\frac{\sqrt{d}}{2}. $$

Here $c_{d}=\frac{2\pi^{d/2}}{\Gamma(d/2)d}$. (1.10) yields that

1.11 $$ \frac{N(B_{r}(\vec{n}))}{N(B_{\frac{r}{\sqrt{d}}}(\vec{n}))}\leq C_{0}\quad\forall r>0. $$

Here $C_{0}>1$ depends only on the dimension *d*. It follows from (1.8)–(1.9) and (1.11) that

1.12 $$ C_{0}^{-m+\frac{\alpha}{d}}\mathfrak{M}_{\alpha}(\vec{f}) (\vec {n}) \leq\mathbf{M}_{\alpha}(\vec{f}) (\vec{n})\leq C_{0}^{m-\frac {\alpha}{d}} \mathfrak{M}_{\alpha}(\vec{f}) (\vec{n}). $$

(1.8), (1.10) and (1.12) imply that

1.13 $$ \mathbf{M}_{\alpha}(\vec{f}) (\vec{n})\leq\widetilde{\mathbf {M}}_{\alpha}(\vec{f}) (\vec{n}) \lesssim_{\alpha,m,d} \mathbf{M}_{\alpha}(\vec{f}) (\vec{n})\lesssim _{\alpha,m,d} \mathfrak{M}_{\alpha}(\vec{f}) (\vec{n})\quad\forall \vec{n}\in \mathbb{Z}^{d}. $$

(1.13) together with the bounds for $\mathfrak{M}_{\alpha}$ leads to (1.7).

Based on the above, a natural question, which arises from the above results, is the following:

#### Question C

Are both $\mathbf{M}_{\alpha}$ and $\widetilde{\mathbf{M}}_{\alpha}$ bounded and continuous from $\ell^{1}(\mathbb{Z}^{d})\times\cdots\times\ell^{1}(\mathbb{Z}^{d})$ to $\operatorname{BV}(\mathbb{Z}^{d})$?

This problem is the main motivation for this work. We will give an affirmative answer by our next theorem.

#### Theorem 1.1

*Let* $d\geq1$ *and* $0\leq\alpha<(m-1)d+1$. *Then* $\mathbf{M}_{\alpha}$ *maps* $\ell^{1}(\mathbb{Z}^{d})\times\cdots\times\ell^{1} (\mathbb{Z}^{d})$ *into* $\operatorname{BV}(\mathbb{Z}^{d})$ *boundedly and continuously*. *Moreover*, *if* $\vec{f}=(f_{1},\ldots,f_{m})$ *with each* $f_{j}\in\ell^{1}(\mathbb{Z}^{d})$, *then*

1.14 $$ \operatorname{Var}\bigl(\mathbf{M}_{\alpha}(\vec{f})\bigr) \lesssim_{\alpha,m,d}\prod_{i=1}^{m} \Vert f_{i} \Vert _{\ell^{1}(\mathbb{Z}^{d})}. $$

*The same results hold for the operator* $\widetilde{\mathbf{M}}_{\alpha}$.

#### Remark 1.1

(i) By (1.4) and Theorem 1.1, we know that both $\vec{f}\mapsto |\nabla\mathbf{M}_{\alpha}(\vec{f})|$ and $\vec{f}\mapsto |\nabla\widetilde{\mathbf{M}}_{\alpha}(\vec{f})|$ are bounded and continuous from $\ell^{1}(\mathbb{Z}^{d}) \times\ell^{1}(\mathbb{Z}^{d})\times\cdots\times\ell^{1} (\mathbb{Z}^{d})$ to $\ell^{1}(\mathbb{Z}^{d})$ if $d\geq1$ and $0\leq\alpha<(m-1)d+1$.

(ii) Both $\mathbf{M}_{\alpha}$ and $\widetilde{\mathbf{M}}_{\alpha}$ are not bounded from $\ell^{1}(\mathbb{Z}^{d})\times\ell^{1}(\mathbb{Z}^{d})\times\cdots \times\ell^{1}(\mathbb{Z}^{d})$ into $\operatorname{BV}(\mathbb{Z}^{d})$ when $(m-1)d+1<\alpha<md$.

(iii) Both $\mathbf{M}_{\alpha}$ and $\widetilde{\mathbf{M}}_{\alpha}$ are not bounded from $\operatorname{BV}(\mathbb{Z}^{d})\times\operatorname{BV} (\mathbb{Z}^{d})\times\cdots\times\operatorname{BV}(\mathbb{Z}^{d})$ into $\operatorname{BV}(\mathbb{Z}^{d})$ when $(m-1)(d-1)<\alpha<md$.

To see the above claims (ii) and (iii), let us only consider the centered case. Let $l\in\mathbb{N}\setminus\{0\}$ with $l>2(\Lambda_{0}+1)$ and $\vec{f}=(f_{1},\ldots,f_{m})$ with each $f_{j}(\vec{n})=\chi_{\{0\leq\Vert\vec{n} \Vert_{1}\leq l\}} (\vec{n})$. Here $\Lambda_{0}$ is given as in (2.3). One can easily check that $\Vert f_{j} \Vert_{\ell^{1}(\mathbb{Z}^{d})}=N_{1,d}(l) \lesssim_{d}l^{d}$, $\Vert\nabla f \Vert_{\ell^{1}(\mathbb{Z}^{d})} \lesssim_{d}l^{d-1}$ and $\mathbf{M}_{\alpha}(\vec{f}) (\vec{n})=(N_{1,d}(l- \Vert\vec{n} \Vert_{1}))^{\frac{\alpha}{d}}$ when $0\leq\Vert\vec{n} \Vert_{1}\leq l$. Then we have

$$\begin{aligned} \bigl\Vert D_{1}\mathbf{M}_{\alpha}(\vec{f}) \bigr\Vert _{\ell ^{1}(\mathbb{Z}^{d})}&\geq\sum_{\Lambda_{0}\leq \Vert (n',n_{d}) \Vert _{1}\leq\frac{l-1}{2}\atop n_{d}\geq0}\bigl( \bigl(N_{1,d}\bigl(l- \bigl\Vert n' \bigr\Vert _{1}- \vert n_{d} \vert \bigr)\bigr)^{\alpha /d}\\ &\quad {}- \bigl(N_{1,d}\bigl(l- \bigl\Vert n' \bigr\Vert _{1}- \vert n_{d} \vert -1\bigr)\bigr)^{\alpha/d} \bigr). \end{aligned} $$

Since $l- \Vert n' \Vert_{1}-|n_{d}|-1>\Lambda_{0}$ when $\Lambda_{0}\leq \Vert(n',n_{d}) \Vert_{1}\leq\frac{l-1}{2}$. Then, by (2.11) with $\gamma=\frac{\alpha}{d}$ and (2.3),

$$\begin{aligned} \bigl\Vert \nabla\mathbf{M}_{\alpha}(\vec{f}) \bigr\Vert _{\ell ^{1}(\mathbb{Z}^{d})}&\gtrsim_{\alpha,d}\sum_{\Lambda_{0}\leq \Vert (n',n_{d}) \Vert _{1}\leq\frac{l-1}{2}\atop n_{d}\geq0} \bigl(l- \bigl\Vert n' \bigr\Vert _{1}- \vert n_{d} \vert -1\bigr)^{\alpha -1}\\ &\gtrsim_{\alpha,d}\biggl( \frac{l-1}{2}\biggr)^{\alpha-1}\biggl(\biggl(\frac {l-1}{2} \biggr)^{d}-\Lambda_{0}^{d}\biggr). \end{aligned} $$

Consequently,

$$\begin{aligned}& \frac{ \Vert \nabla\mathbf{M}_{\alpha}(\vec{f}) \Vert _{\ell^{1}(\mathbb{Z}^{d})}}{\prod_{j=1}^{m} \Vert f_{j} \Vert _{\ell^{1}(\mathbb{Z}^{d})}} \gtrsim_{\alpha,m,d}\frac{l^{\alpha+d-1}-\Lambda_{0}^{d}l^{\alpha -1}}{l^{md}}\gtrsim_{\alpha,m,d}l^{\alpha+d-1-md}- \Lambda _{0}^{d}l^{\alpha-1-md}, \\& \frac{ \Vert \nabla\mathbf{M}_{\alpha}(\vec{f}) \Vert _{\ell^{1}(\mathbb{Z}^{d})}}{\prod_{j=1}^{m} \Vert \nabla f_{j} \Vert _{\ell^{1}(\mathbb{Z}^{d})}}\gtrsim_{\alpha,m,d}\frac{l^{\alpha +d-1}-\Lambda_{0}^{d}l^{\alpha-1}}{l^{m(d-1)}}\gtrsim_{\alpha ,m,d}l^{\alpha-(m-1)(d-1)}- \Lambda_{0}^{d}l^{\alpha-1-m(d-1)}. \end{aligned}$$

Letting $l\rightarrow+\infty$, the claims (ii) and (iii) follow.

#### Remark 1.2

It should be pointed out that our main results are new even in the special case $m=1$ and $\alpha=0$.

The rest of this paper is organized as follows. Section 2 contains some notation and necessary lemmas. The proof of Theorem 1.1 is given in Section 3. It should be pointed out that the main method employed in this paper is a combination of ideas and arguments from [18, 33], but our methods and techniques in the proof of Theorem 1.1 are more simple, direct and different than those in [18, 33]. In particular, the proof of Theorem B is highly dependent on a summability argument over the sequence of local maximal and local minima of discrete multisublinear fractional maximal functions and two summability estimates (see [33, Lemmas 2.1–2.2]). In [18, 33], the proofs of the corresponding continuity results are highly dependent on the Brezis–Lieb lemma [35]. Moreover, the discrete versions of Luiro’s lemma (see [18, Lemmas 4–5]) have also played key roles in the proof of the corresponding continuity results in [18]. However, these tools and lemmas are unnecessary in our proof. We would like to remark that the proposed method in this paper can be extended to study the convergence of the parameter estimation algorithms for linear and bilinear systems (see [36–38]). Throughout this paper, the letter *C* will denote a positive constant that may vary at each occurrence but is independent of the essential variables. If there exists a constant $C>0$ depending only on *ϑ* such that $A\leq CB$, we then write $A\lesssim_{\vartheta}B$ or $B\gtrsim_{\vartheta}A$; and if $A\lesssim_{\vartheta}B\lesssim_{\vartheta}A$, we then write $A\thicksim_{\vartheta}B$. We also use the conventions $\prod_{i\in\emptyset}a_{i}=1$ and $\sum_{i\in\emptyset}a_{i}=0$.

## Preliminary notations and lemmas

Let $\mathbb{N}=\{0,1,2,\ldots\}$. For any $r\in\mathbb{N}$, we denote by $N_{1,d}(r)$ the number of elements in the set $\{\vec{n}=(n_{1},\ldots,n_{d})\in \mathbb{Z}^{d}: \Vert\vec{n} \Vert_{1}\leq r\}$. It is obvious that $N_{1,d}(0)=1$ and $N_{1,d}(r+1)>N_{1,d}(r)\geq1$ for all $r\in\mathbb{N}$. Fix $\vec{n}\in\mathbb{Z}^{d}$, since $\Vert\vec{n} \Vert_{2}\leq\Vert\vec{n} \Vert_{1}\leq\sqrt{d} \Vert\vec{n} \Vert_{2}$, then

2.1 $$ N\bigl(B_{\frac{r-1}{\sqrt{d}}}(\vec{0})\bigr)\leq N_{1,d}(r)\leq N \bigl(B_{r+1}(\vec{0})\bigr) \quad\forall r\in\mathbb{N}\setminus\{0,1\} . $$

(2.1) and (1.10) give that

2.2 $$ c_{d}\biggl(\frac{r-1}{\sqrt{d}}-\frac{\sqrt{d}}{2}\biggr)^{d}_{+} \leq N_{1,d}(r)\leq c_{d}\biggl(r+\frac{\sqrt{d}}{2}+1 \biggr)^{d} \quad\forall r\in \mathbb{N}\setminus\{0,1\}. $$

Here $c_{d}$ is given as in (1.10) and $r_{+}:=\max\{r, c_{d}^{-1/d}\}$ for any $r>0$. By (2.2), there exists $\Lambda_{0}\in\mathbb{N}\setminus\{0\}$ such that

2.3 $$\begin{aligned}& N_{1,d}(r)\thicksim_{d}r^{d}\quad\forall r\geq \Lambda_{0}; \end{aligned}$$

2.4 $$\begin{aligned}& N_{1,d}(r+1)-N_{1,d}(r)\thicksim_{d}r^{d-1} \quad\forall r\geq\Lambda _{0}. \end{aligned}$$

The following lemmas will play key roles in the proof of Theorem 1.1.

### Lemma 2.1

*Let* $\gamma>0$, $d\geq1$ *and* $\Lambda_{0}$ *is given as in* (2.3). *Define the function* $\Phi_{\gamma}:\mathbb{N}\rightarrow\mathbb{R}$ *by* $\Phi_{\gamma}(r)=(N_{1,d}(r))^{-\gamma}-(N_{1,d}(r+1))^{-\gamma}$. *Then*

(i) $\Phi_{\gamma}$ *is strictly decreasing on* $\mathbb{N}$.
(ii) $\Phi_{\gamma}(r)\sim_{\gamma,d,\Lambda_{0}}\Phi(2r)$ *for any* $r\in\mathbb{N}$.
(iii) $\Phi_{\gamma}(r)\sim_{\gamma,d,\Lambda_{0}}(N_{1,d} (r+1))^{-\gamma-\frac{1}{d}}$ *for any* $r\in\mathbb{N}$.

### Proof

When $d=1$. It is obvious that $\Phi_{\gamma}(r)=(2r+1)^{-\gamma}-(2r+3)^{-\gamma}$ is strictly decreasing on $r\in\mathbb{N}$. To prove (i) for the case $d\geq2$, it suffices to show that

2.5 $$ \Phi_{\gamma}(r)>\Phi_{\gamma}(r+1)\quad\forall r\in\mathbb {N}. $$

(2.5) reduces to the following:

2.6 $$ \frac{(N_{1,d}(r+1))^{\gamma}}{(N_{1,d}(r))^{\gamma}}+\frac {(N_{1,d}(r+1))^{\gamma}}{(N_{1,d}(r+2))^{\gamma}}>2\quad\forall r\in \mathbb{N}. $$

It was shown in [28, Lemma 4] that

2.7 $$ \frac{N_{1,d}(r+1)}{N_{1,d}(r)}>\frac {N_{1,d}(r+2)}{N_{1,d}(r+1)}\quad\forall r\in\mathbb{N}. $$

Combining (2.7) with the arithmetic mean-geometric mean inequality yields (2.6).

To prove (ii), it suffices to show that

2.8 $$ \bigl(N_{1,d}(r)\bigr)^{-\gamma}-\bigl(N_{1,d}(r+1) \bigr)^{-\gamma}\sim_{\gamma ,d,\Lambda_{0}}(r+1)^{-1-d\gamma}\quad\forall r\in \mathbb{N}. $$

Let us begin with proving the following:

2.9 $$ \bigl(N_{1,d}(r+1)\bigr)^{\gamma}-\bigl(N_{1,d}(r) \bigr)^{\gamma}\thicksim_{\gamma ,d}r^{d\gamma-1}\quad\forall r\geq \Lambda_{0}. $$

We consider the following two cases.

*Case A*. $\gamma\in\mathbb{N}\setminus\{0\}$. When $\gamma=1$, (2.9) is obvious by (2.4). When $\gamma\geq2$, we have

$$\begin{aligned}[b] &\bigl(N_{1,d}(r+1)\bigr)^{\gamma}- \bigl(N_{1,d}(r)\bigr)^{\gamma}\\ &\quad=\bigl(N_{1,d}(r+1)-N_{1,d}(r)\bigr) \bigl(N_{1,d}(r+1)^{\gamma -1}+N_{1,d}(r+1)^{\gamma-2}N_{1,d}(r)+ \cdots+\bigl(N_{1,d}(r)\bigr)^{\gamma -1} \bigr). \end{aligned} $$

This together with (2.3) and (2.4) yields (2.9) for the case $\gamma\geq2$.

*Case B*. $\gamma\notin\mathbb{N}$. We can write $\gamma=\frac{p}{q}$ for some $p,q\in\mathbb{N}$ with $p\geq1$ and $q\geq2$. Observe that

$$a-b=\bigl(a^{\frac{1}{n}}\bigr)^{n}-\bigl(b^{\frac{1}{n}} \bigr)^{n}=\bigl(a^{\frac {1}{n}}-b^{\frac{1}{n}}\bigr) \bigl(a^{\frac{n-1}{n}}+a^{\frac{n-2}{n}}b^{\frac {1}{n}}+\cdots+a^{\frac{1}{n}}b^{\frac{n-2}{n}}+b^{\frac{n-1}{n}} \bigr) $$

for any $a,b>0$. It follows that

$$a^{\frac{1}{n}}-b^{\frac{1}{n}}=\frac{a-b}{a^{\frac {n-1}{n}}+a^{\frac{n-2}{n}}b^{\frac{1}{n}}+\cdots+a^{\frac {1}{n}}b^{\frac{n-2}{n}}+b^{\frac{n-1}{n}}} $$

for any $a,b>0$. Then

2.10 $$ a^{\gamma}-b^{\gamma}=\bigl(a^{p}\bigr)^{\frac{1}{q}}- \bigl(b^{p}\bigr)^{\frac{1}{q}}=\frac {a^{p}-b^{p}}{a^{\frac{p(q-1)}{q}}+a^{\frac{p(q-2)}{q}}b^{\frac{p}{q}} +\cdots+a^{\frac{p}{q}}b^{\frac{p(q-2)}{q}}+b^{\frac {p(q-1)}{q}}} $$

for any $a,b>0$. From (2.3), (2.10) and Case A with $\gamma=p$, we have

$$\bigl(N_{1,d}(r+1)\bigr)^{\gamma}-\bigl(N_{1,d}(r) \bigr)^{\gamma}\sim_{\gamma,d}r^{dp-1} q^{-1}r^{-d\frac{p(q-1)}{q}} \thicksim_{\gamma,d}r^{d\gamma-1}\quad \forall r\geq\Lambda_{0}, $$

which establishes (2.9) in this case.

It follows from (2.3) and (2.9) that

2.11 $$ \begin{aligned}[b] \bigl(N_{1,d}(r)\bigr)^{-\gamma}-\bigl(N_{1,d}(r+1) \bigr)^{-\gamma}&=\frac {(N_{1,d}(r+1))^{\gamma}-(N_{1,d}(r))^{\gamma}}{(N_{1,d}(l+1))^{\gamma}(N_{1,d}(r))^{\gamma}}\\ &\sim_{\gamma,d}(r+1)^{-1-d\gamma} \quad\forall r\geq\Lambda_{0}. \end{aligned} $$

When $0\leq r<\Lambda_{0}$. By (i) and (2.11), we get

$$\begin{aligned} \bigl(N_{1,d}(r)\bigr)^{-\gamma}-\bigl(N_{1,d}(r+1) \bigr)^{-\gamma}&\geq\bigl(N_{1,d}(\Lambda _{0}) \bigr)^{-\gamma}-\bigl(N_{1,d}(\Lambda_{0}+1) \bigr)^{-\gamma}\\ &\gtrsim_{\gamma ,d}(\Lambda_{0}+1)^{-1-d\gamma} \gtrsim_{\gamma,d,\Lambda _{0}}(r+1)^{-1-d\gamma}. \end{aligned} $$

This together with the trivial inequality $(N_{1,d} (r))^{-\gamma}-(N_{1,d}(r+1))^{-\gamma}\lesssim_{\gamma,d, \Lambda_{0}}(r+1)^{-1-d\gamma}$ for any $0\leq r<\Lambda_{0}$ yields that

2.12 $$ \bigl(N_{1,d}(r)\bigr)^{-\gamma}-\bigl(N_{1,d}(r+1) \bigr)^{-\gamma}\sim_{\gamma ,d,\Lambda_{0}}(r+1)^{-1-d\gamma}\quad\forall0\leq r< \Lambda _{0}. $$

Combining (2.12) with (2.11) yields (2.8).

It remains to prove (iii). By (2.3) and (2.8), we get

2.13 $$ \Phi_{\gamma}(r)\sim_{\gamma,d}\bigl(N_{1,d}(r+1) \bigr)^{-\gamma-\frac {1}{d}}\quad\forall r\geq\Lambda_{0}. $$

On the other hand, we get from (2.2) that

$$\bigl(N_{1,d}(r+1)\bigr)^{-\gamma-\frac{1}{d}}\gtrsim_{\gamma ,d}(r+1)^{-1-d\gamma} \quad\forall0\leq r< \Lambda_{0}. $$

This together with (2.10) and the trivial fact that $(N_{1,d}(r+1))^{-\gamma-\frac{1}{d}}\leq1 \lesssim_{\gamma,d,\Lambda_{0}}(r+1)^{-1-d\gamma}$ for $0\leq r<\Lambda_{0}$ implies that

$$\bigl(N_{1,d}(r+1)\bigr)^{-\gamma-\frac{1}{d}}\sim_{\gamma,d,\Lambda_{0}}\Phi _{\gamma}(r)\quad\forall0\leq r< \Lambda_{0}, $$

which together with (2.13) yields (iii). □

### Lemma 2.2

*Let* $d\geq2$, $\gamma>1$ *and* $R\in\mathbb{N}$ *with* $R\geq\Lambda_{0}$. *Then*

2.14 $$\begin{aligned}& \sum_{ \Vert \vec{n} \Vert _{1}\geq R}\bigl(N_{1,d}\bigl( \Vert \vec{n} \Vert _{1}\bigr)\bigr)^{-\gamma}\lesssim_{\gamma ,d}R^{d-d\gamma}; \end{aligned}$$

2.15 $$\begin{aligned}& \sum_{\vec{n}\in\mathbb{Z}^{d}}\bigl(N_{1,d}\bigl( \Vert \vec{n} \Vert _{1}\bigr)\bigr)^{-\gamma}\lesssim_{\gamma,d}1. \end{aligned}$$

### Proof

We only prove (2.14), since (2.15) follows from (2.14) and the following:

$$\sum_{ \Vert \vec{n} \Vert _{1}< \Lambda_{0}}\bigl(N_{1,d}\bigl( \Vert \vec{n} \Vert _{1}\bigr)\bigr)^{-\gamma}\leq N_{1,d}( \Lambda _{0})\lesssim_{d}\Lambda_{0}^{d}, $$

where in the last inequality of the above inequality we have used (2.3). For $s\in\mathbb{N}$, let $r_{1,d}(s)$ denote the number of elements in the set $\{\vec{n}=(n_{1},n_{2}, \ldots,n_{d})\in\mathbb{Z}^{d}: \Vert\vec{n} \Vert_{1}=s\}$. Since $d-d\gamma<0$, then by (2.3) and (2.4) we have

$$\begin{aligned} \sum_{ \Vert \vec{n} \Vert _{1}\geq R}\bigl(N_{1,d}\bigl( \Vert \vec{n} \Vert _{1}\bigr)\bigr)^{-\gamma} &=\sum _{l=R}^{+\infty }\bigl(N_{1,d}(l) \bigr)^{-\gamma}r_{1,d}(l) \lesssim_{\gamma,d}\sum _{l=R}^{+\infty}l^{-d\gamma }\bigl(N_{1,d}(l)-N_{1,d}(l-1) \bigr) \\ &\lesssim_{\gamma,d}\sum_{l=R}^{+\infty}l^{-d\gamma}(l-1)^{d-1} \lesssim_{\gamma,d}\sum_{l=R}^{+\infty}l^{d-1-d\gamma} \lesssim _{\gamma,d}R^{d-d\gamma}, \end{aligned}$$

which gives (2.14) and completes the proof of Lemma 2.2. □

## Proof of Theorem 1.1

This section is devoted to the proof of Theorem 1.1. Let $\Phi_{\gamma}$ be defined as in Lemma 2.1. It is clear that

3.1 $$ \bigl(N\bigl(\Gamma_{r}(\vec{n})\bigr)\bigr)^{\frac{\alpha}{d}-m}-\bigl(N \bigl(\Gamma_{r+1}(\vec {n})\bigr)\bigr)^{\frac{\alpha}{d}-m}= \Phi_{m-\frac{\alpha}{d}}\bigl([r]\bigr)\quad \forall\vec{n}\in\mathbb{Z}^{d} \mbox{ and } r\in[0,\infty). $$

### Proof of Theorem 1.1—boundedness part

Let $\vec{f}=(f_{1},\ldots,f_{m})$ with each $f_{j}\in\ell^{1} (\mathbb{Z}^{d})$. Without loss of generality, we assume that all $f_{j}\geq0$. We divide the proof of this part into two cases.

#### Centered case

To prove (1.14), it suffices to show that

3.2 $$ \bigl\Vert D_{l}\mathbf{M}_{\alpha}(\vec{f}) \bigr\Vert _{\ell ^{1}(\mathbb{Z}^{d})} \lesssim_{\alpha,m,d}\prod_{i=1}^{m} \Vert f_{i} \Vert _{\ell^{1}(\mathbb{Z}^{d})} $$

for all $1\leq l\leq d$. We shall work with (3.2) for $l=d$ and the other cases are analogous. In what follows, we set $\vec{n}=(n',n_{d})\in\mathbb{Z}^{d}$ with $n'=(n_{1}, \ldots,n_{d-1})\in\mathbb{Z}^{d-1}$. It is clear that

$$\bigl\Vert D_{d}\mathbf{M}_{\alpha}(\vec{f}) \bigr\Vert _{\ell ^{1}(\mathbb{Z}^{d})} =\sum_{n'\in\mathbb{Z}^{d-1}}\sum _{n_{d}\in\mathbb{Z}} \bigl\vert \mathbf{M}_{\alpha}(\vec{f}) \bigl(n',n_{d}+1\bigr)-\mathbf{M}_{\alpha}(\vec{f}) \bigl(n',n_{d}\bigr) \bigr\vert . $$

For each $n'\in\mathbb{Z}^{d-1}$, let

$$\begin{aligned}& X_{n'}=\bigl\{ n_{d}\in\mathbb{Z}: \mathbf{M}_{\alpha}( \vec {f}) \bigl(n',n_{d}+1\bigr)=\mathbf{M}_{\alpha}( \vec{f}) \bigl(n',n_{d}\bigr)\bigr\} . \\& X_{n'}^{+}=\bigl\{ n_{d}\in\mathbb{Z}: \mathbf{M}_{\alpha}(\vec {f}) \bigl(n',n_{d}+1 \bigr)>\mathbf{M}_{\alpha}(\vec{f}) \bigl(n',n_{d} \bigr)\bigr\} , \\& X_{n'}^{-}=\bigl\{ n_{d}\in\mathbb{Z}: \mathbf{M}_{\alpha}(\vec {f}) \bigl(n',n_{d}+1 \bigr)< \mathbf{M}_{\alpha}(\vec{f}) \bigl(n',n_{d} \bigr)\bigr\} . \end{aligned}$$

Then we can write

$$\begin{aligned}[b] \bigl\Vert D_{d}\mathbf{M}_{\alpha}( \vec{f}) \bigr\Vert _{\ell ^{1}(\mathbb{Z}^{d})} &=\sum_{n'\in\mathbb{Z}^{d-1}} \sum_{n_{d}\in X_{n'}^{+}}\bigl(\mathbf {M}_{\alpha}(\vec{f}) \bigl(n',n_{d}+1\bigr)-\mathbf{M}_{\alpha}(\vec{f}) \bigl(n',n_{d}\bigr)\bigr) \\ &\quad{}+\sum_{n'\in\mathbb{Z}^{d-1}}\sum _{n_{d}\in X_{n'}^{-}}\bigl(\mathbf{M}_{\alpha}(\vec{f}) \bigl(n',n_{d}\bigr)-\mathbf{M}_{\alpha}(\vec{f}) \bigl(n',n_{d}+1\bigr)\bigr). \end{aligned} $$

Thus, to prove (3.2), it suffices to show that

3.3 $$\begin{aligned}& \sum_{n'\in\mathbb{Z}^{d-1}}\sum_{n_{d}\in X_{n'}^{+}} \bigl(\mathbf {M}_{\alpha}(\vec{f}) \bigl(n',n_{d}+1 \bigr)-\mathbf{M}_{\alpha}(\vec {f}) \bigl(n',n_{d} \bigr)\bigr)\lesssim_{\alpha,m,d}\prod_{i=1}^{m} \Vert f_{i} \Vert _{\ell^{1}(\mathbb{Z}^{d})}; \end{aligned}$$

3.4 $$\begin{aligned}& \sum_{n'\in\mathbb{Z}^{d-1}}\sum_{n_{d}\in X_{n'}^{-}} \bigl(\mathbf {M}_{\alpha}(\vec{f}) \bigl(n',n_{d} \bigr)-\mathbf{M}_{\alpha}(\vec {f}) \bigl(n',n_{d}+1 \bigr)\bigr)\lesssim_{\alpha,m,d}\prod_{i=1}^{m} \Vert f_{i} \Vert _{\ell^{1}(\mathbb{Z}^{d})}. \end{aligned}$$

We only prove (3.3) since (3.4) can be obtained similarly. For $r\in\mathbb{N}$, we define the function $A_{r}(\vec{f}): \mathbb{Z}^{d}\rightarrow\mathbb{R}$ by

$$A_{r}(\vec{f}) (\vec{n})=\bigl(N\bigl(\Gamma_{r}(\vec{n}) \bigr)\bigr)^{\frac{\alpha }{d}-m}\prod_{j=1}^{m} \sum_{\vec{k}\in\Gamma_{r}(\vec{n})}f_{j}(\vec {k})\quad\forall \vec{n}\in\mathbb{Z}^{d}. $$

Since all $f_{j}\in\ell^{1}(\mathbb{Z}^{d})$, then $\lim_{r\rightarrow\infty}A_{r}(\vec{f})(\vec{n})=0$. It follows that for any $n'\in\mathbb{Z}^{d-1}$ and $n_{d} \in X_{n'}^{+}$, there exists $r(n',n_{d})>0$ such that $\mathbf{M}_{\alpha}(\vec{f})(n',n_{d})=A_{r(n',n_{d})} (\vec{f})(n',n_{d})$. This together with (3.1) yields that

3.5 $$ \begin{aligned}[b] &\mathbf{M}_{\alpha}(\vec{f}) \bigl(n',n_{d}+1\bigr)-\mathbf{M}_{\alpha}(\vec {f}) \bigl(n',n_{d}\bigr) \\ &\quad\leq A_{r(n',n_{d}+1)}(\vec{f}) \bigl(n',n_{d}+1 \bigr)-A_{r(n',n_{d}+1)+1}(\vec {f}) \bigl(n',n_{d}\bigr) \\ &\quad\leq\Phi\bigl(\bigl[r\bigl(n',n_{d}+1\bigr)\bigr] \bigr)\prod_{j=1}^{m}\sum _{\vec{k}\in\Gamma _{r(n',n_{d}+1)}(n',n_{d}+1)}f_{j}(\vec{k}) \\ &\quad\leq\Biggl(\prod_{i=1}^{m-1} \Vert f_{i} \Vert _{\ell ^{1}(\mathbb{Z}^{d})}\Biggr)\sum_{\vec{k}\in\Gamma _{r(n',n_{d}+1)}(n',n_{d}+1)} \Phi_{m-\frac{\alpha }{d}}\bigl(\bigl[r\bigl(n',n_{d}+1\bigr) \bigr]\bigr)f_{m}(\vec{k}). \end{aligned} $$

It follows that

3.6 $$ \begin{aligned}[b] &\sum_{n'\in\mathbb{Z}^{d-1}}\sum _{n_{d}\in X_{n'}^{+}}\bigl(\mathbf {M}_{\alpha}(\vec{f}) \bigl(n',n_{d}+1\bigr)-\mathbf{M}_{\alpha}(\vec{f}) \bigl(n',n_{d}\bigr)\bigr) \\ &\quad\leq\Biggl(\prod_{i=1}^{m-1} \Vert f_{i} \Vert _{\ell ^{1}(\mathbb{Z}^{d})}\Biggr)\sum_{n'\in\mathbb{Z}^{d-1}} \sum_{n_{d}\in X_{n'}^{+}}\sum_{\vec{k}\in\Gamma_{r(n',n_{d}+1)}(n',n_{d}+1)}\Phi _{m-\frac{\alpha}{d}}\bigl(\bigl[r\bigl(n',n_{d}+1\bigr)\bigr] \bigr)f_{m}(\vec{k}) \\ &\quad\leq\Biggl(\prod_{i=1}^{m-1} \Vert f_{i} \Vert _{\ell ^{1}(\mathbb{Z}^{d})}\Biggr)\sum_{\vec{k}\in\mathbb{Z}^{d}}f_{m}(\vec{k}) \\ &\qquad{}\times \sum_{n'\in\mathbb{Z}^{d-1}}\sum_{n_{d}\in X_{n'}^{+}} \Phi_{m-\frac {\alpha}{d}}\bigl(\bigl[r\bigl(n',n_{d}+1\bigr) \bigr]\bigr)\chi_{\{ \Vert \vec{k}-(n',n_{d}+1) \Vert _{1}\leq[r(n',n_{d}+1)]\}}\bigl(n',n_{d}\bigr). \end{aligned} $$

Fix $\vec{k}\in\mathbb{Z}^{d}$. Invoking Lemmas 2.1–2.2, we have

3.7 $$ \begin{aligned}[b] &\sum_{n'\in\mathbb{Z}^{d-1}}\sum _{n_{d}\in X_{n'}^{+}}\Phi_{m-\frac {\alpha}{d}}\bigl(\bigl[r \bigl(n',n_{d}+1\bigr)\bigr]\bigr)\chi_{\{ \Vert \vec{k}-(n',n_{d}+1) \Vert _{1}\leq[r(n',n_{d}+1)]\}} \bigl(n',n_{d}\bigr) \\ &\quad\leq\sum_{n'\in\mathbb{Z}^{d-1}}\sum _{n_{d}\in X_{n'}^{+}}\Phi_{m-\frac{\alpha}{d}}\bigl( \bigl\Vert \vec {k}- \bigl(n',n_{d}+1\bigr) \bigr\Vert _{1}\bigr) \\ &\quad\leq\sum_{\vec{n}\in\mathbb{Z}^{d}}\Phi_{m-\frac{\alpha }{d}}\bigl( \Vert \vec{n} \Vert _{1}\bigr) \lesssim_{\alpha,m,d}\sum _{\vec{n}\in\mathbb{Z}^{d}}\bigl(N_{1,d}\bigl( \Vert \vec{n} \Vert _{1}\bigr)\bigr)^{\frac{\alpha-1}{d}-m}\lesssim _{\alpha,m,d}1. \end{aligned} $$

In the last inequality of (3.7) we have used the fact $\alpha<(m-1)d+1$. Then (3.3) follows from (3.6) and (3.7).

#### Uncentered case

In this case the arguments are similar to those in the centered case, but the arguments are more complex than those in the centered case. We want to show that

3.8 $$ \bigl\Vert D_{d}\widetilde{\mathbf{M}}_{\alpha}(\vec{f}) \bigr\Vert _{\ell^{1}(\mathbb{Z}^{d})} \lesssim_{\alpha,m,d}\prod _{i=1}^{m} \Vert f_{i} \Vert _{\ell^{1}(\mathbb{Z}^{d})}. $$

For each $n'\in\mathbb{Z}^{d-1}$, let

$$\begin{aligned}& Y_{n'}=\bigl\{ n_{d}\in\mathbb{Z}: \widetilde{ \mathbf{M}}_{\alpha}(\vec {f}) \bigl(n',n_{d}+1 \bigr)=\widetilde{\mathbf{M}}_{\alpha}(\vec{f}) \bigl(n',n_{d} \bigr)\bigr\} , \\& Y_{n'}^{+}=\bigl\{ n_{d}\in\mathbb{Z}: \widetilde{\mathbf{M}}_{\alpha}(\vec {f}) \bigl(n',n_{d}+1 \bigr)>\widetilde{\mathbf{M}}_{\alpha}(\vec{f}) \bigl(n',n_{d} \bigr)\bigr\} , \\& Y_{n'}^{-}=\bigl\{ n_{d}\in\mathbb{Z}: \widetilde{\mathbf{M}}_{\alpha}(\vec {f}) \bigl(n',n_{d}+1 \bigr)< \widetilde{\mathbf{M}}_{\alpha}(\vec{f}) \bigl(n',n_{d} \bigr)\bigr\} . \end{aligned}$$

Fix $n'\in\mathbb{Z}^{d-1}$. Since all $f_{j}\in\ell^{1} (\mathbb{Z}^{d})$, then for any $n_{d}\in Y_{n'}^{+}$, there exist $r(n',n_{d}+1)>0$ and $\vec{l}\in \mathbb{Z}^{d}$ such that $\widetilde{\mathbf{M}}_{\alpha}(\vec{f})(n',n_{d}+1)=A_{r(n',n_{d}+1)}(\vec{f})(\vec{l})$ and $\Vert(n',n_{d}+1)-\vec{l} \Vert_{1}< r(n',n_{d}+1)$. By the arguments similar to those used in deriving (3.5), we obtain

3.9 $$ \begin{aligned}[b] &\widetilde{\mathbf{M}}_{\alpha}(\vec{f}) \bigl(n',n_{d}+1\bigr)-\widetilde {\mathbf{M}}_{\alpha}( \vec{f}) \bigl(n',n_{d}\bigr) \\ &\quad\leq A_{r(n',n_{d}+1)}(\vec{f}) (\vec{l})-A_{r(n',n_{d}+1)+1}(\vec {f}) ( \vec{l}-\vec{e}_{d}) \\ &\quad\leq\Phi\bigl(\bigl[r\bigl(n',n_{d}+1\bigr)\bigr] \bigr)\prod_{j=1}^{m}\sum _{\vec{k}\in\Gamma _{r(n',n_{d}+1)}(\vec{l})}f_{j}(\vec{k}) \\ &\quad\leq\Biggl(\prod_{i=1}^{m-1} \Vert f_{i} \Vert _{\ell ^{1}(\mathbb{Z}^{d})}\Biggr)\sum_{\vec{k}\in\Gamma _{2r(n',n_{d}+1)}(n',n_{d}+1)} \Phi_{m-\frac{\alpha }{d}}\bigl(\bigl[r\bigl(n',n_{d}+1\bigr) \bigr]\bigr)f_{m}(\vec{k}). \end{aligned} $$

Note that $8[r]\geq[2r]$ for $r\geq2$ and $\Phi(r)\leq1$ for all $r\in\mathbb{N}$. By Lemma 2.1, one can get that

3.10 $$\begin{aligned} &\sum_{\vec{k}\in\Gamma_{2r(n',n_{d}+1)}(n',n_{d}+1)} \Phi_{m-\frac {\alpha}{d}}\bigl(\bigl[r\bigl(n',n_{d}+1\bigr) \bigr]\bigr)f_{m}(\vec{k}) \\ &\quad\lesssim_{\alpha,m,d}\sum_{\vec{k}\in\Gamma _{2r(n',n_{d}+1)}(n',n_{d}+1)} \Phi_{m-\frac{\alpha }{d}}\bigl(8\bigl[r\bigl(n',n_{d}+1\bigr) \bigr]\bigr)f_{m}(\vec{k}) \\ &\quad\lesssim_{\alpha,m,d}\sum_{\vec{k}\in\Gamma _{2r(n',n_{d}+1)}(n',n_{d}+1)}f_{m}( \vec{k})\chi_{\{ \Vert \vec {k}-(n',n_{d}+1) \Vert _{1}< 2r(n',n_{d}+1)< 4\}}\bigl(n',n_{d}\bigr) \\ &\hphantom{\quad\lesssim_{\alpha,m,d}}{}+\sum_{\vec{k}\in\Gamma_{2r(n',n_{d}+1)}(n',n_{d}+1)}\Phi _{m-\frac{\alpha}{d}}\bigl( \bigl[2r\bigl(n',n_{d}+1\bigr)\bigr]\bigr)f_{m}( \vec{k})\chi_{\{ r(n',n_{d}+1)\geq2\}}\bigl(n',n_{d}\bigr) \\ &\quad\lesssim_{\alpha,m,d}\sum_{\vec{k}\in\mathbb{Z}^{d}}f_{m}( \vec {k})\chi_{\{ \Vert \vec{k}-(n',n_{d}+1) \Vert _{1}< 4\} }\bigl(n',n_{d}\bigr) \\ &\hphantom{\quad\lesssim_{\alpha,m,d}}{}+\sum_{\vec{k}\in\mathbb{Z}^{d}}f_{m}(\vec{k}) \Phi_{m-\frac {\alpha}{d}}\bigl(\bigl[2r\bigl(n',n_{d}+1\bigr) \bigr]\bigr)\chi_{\{ \Vert \vec{k}-(n',n_{d}+1) \Vert _{1}\leq[2r(n',n_{d}+1)]\}}\bigl(n',n_{d}\bigr). \end{aligned}$$

By the arguments similar to those used to derive (3.7), we get

3.11 $$ \begin{aligned}[b] &\sup_{\vec{k}\in\mathbb{Z}^{d}}\sum_{n'\in\mathbb{Z}^{d-1}}\sum _{n_{d}\in X_{n'}^{+}}\Phi_{m-\frac{\alpha}{d}}\bigl(\bigl[2r \bigl(n',n_{d}+1\bigr)\bigr]\bigr)\chi _{\{ \Vert \vec{k}-(n',n_{d}+1) \Vert _{1}\leq [2r(n',n_{d}+1)]\}} \bigl(n',n_{d}\bigr)\\ &\quad \lesssim_{\alpha,m,d}1. \end{aligned} $$

It follows from (3.9)–(3.11) that

3.12 $$ \begin{aligned}[b] &\sum_{n'\in\mathbb{Z}^{d-1}}\sum _{n_{d}\in Y_{n'}^{+}}\bigl(\widetilde {\mathbf{M}}_{\alpha}( \vec{f}) \bigl(n',n_{d}+1\bigr)-\widetilde{\mathbf {M}}_{\alpha}(\vec{f}) \bigl(n',n_{d}\bigr)\bigr) \\ &\quad\lesssim_{\alpha,m,d}\Biggl(\prod_{i=1}^{m-1} \Vert f_{i} \Vert _{\ell^{1}(\mathbb{Z}^{d})}\Biggr)\\ &\hphantom{\quad\lesssim_{\alpha,m,d}} {}\times \biggl(\sum _{n'\in\mathbb {Z}^{d-1}}\sum_{n_{d}\in Y_{n'}^{+}}\sum _{\vec{k}\in\Gamma _{2r(n',n_{d}+1)}(n',n_{d}+1)}f_{m}(\vec{k})\chi_{\{ \Vert \vec {k}-(n',n_{d}+1) \Vert _{1}< 4\}} \bigl(n',n_{d}\bigr) \\ &\hphantom{\quad\lesssim_{\alpha,m,d}}{}+\sum_{n'\in\mathbb{Z}^{d-1}}\sum _{n_{d}\in Y_{n'}^{+}}\sum_{\vec{k}\in\mathbb{Z}^{d}}f_{m}( \vec{k})\Phi_{m-\frac{\alpha }{d}}\bigl(\bigl[2r\bigl(n',n_{d}+1 \bigr)\bigr]\bigr)\\ &\hphantom{\quad\lesssim_{\alpha,m,d}} {}\times\chi_{\{ \Vert \vec{k}-(n',n_{d}+1) \Vert _{1}\leq[2r(n',n_{d}+1)]\}}\bigl(n',n_{d}\bigr) \biggr) \\ &\quad\lesssim_{\alpha,m,d}\Biggl(\prod_{i=1}^{m} \Vert f_{i} \Vert _{\ell^{1}(\mathbb{Z}^{d})}\Biggr) \biggl(\sup _{\vec{k}\in\mathbb{Z}^{d}}\sum_{n'\in\mathbb{Z}^{d-1}}\sum _{n_{d}\in Y_{n'}^{+}}\chi_{\{ \Vert \vec{k}-(n',n_{d}+1) \Vert _{1}< 4\}}\bigl(n',n_{d} \bigr) \\ &\hphantom{\quad\lesssim_{\alpha,m,d}}{}+\sup_{\vec{k}\in\mathbb{Z}^{d}}\sum_{n'\in\mathbb {Z}^{d-1}} \sum_{n_{d}\in Y_{n'}^{+}}\Phi_{m-\frac{\alpha }{d}}\bigl(\bigl[2r \bigl(n',n_{d}+1\bigr)\bigr]\bigr)\\ &\hphantom{\quad\lesssim_{\alpha,m,d}}{}\times\chi_{\{ \Vert \vec{k}-(n',n_{d}+1) \Vert _{1}\leq[2r(n',n_{d}+1)]\}} \bigl(n',n_{d}\bigr)\biggr) \\ &\quad\lesssim_{\alpha,m,d}\prod_{i=1}^{m} \Vert f_{i} \Vert _{\ell^{1}(\mathbb{Z}^{d})}. \end{aligned} $$

Similarly, we can obtain

3.13 $$ \sum_{n'\in\mathbb{Z}^{d-1}}\sum_{n_{d}\in Y_{n'}^{-}} \bigl(\widetilde {\mathbf{M}}_{\alpha}(\vec{f}) \bigl(n',n_{d} \bigr)-\widetilde{\mathbf{M}}_{\alpha}(\vec{f}) \bigl(n',n_{d}+1 \bigr)\bigr)\lesssim_{\alpha,m,d}\prod_{i=1}^{m} \Vert f_{i} \Vert _{\ell^{1}(\mathbb{Z}^{d})}. $$

It follows from (3.12) and (3.13) that

$$\begin{aligned}[b] \bigl\Vert D_{d}\widetilde{ \mathbf{M}}_{\alpha}(\vec{f}) \bigr\Vert _{\ell^{1}(\mathbb{Z}^{d})}&=\sum _{n'\in\mathbb{Z}^{d-1}}\sum_{n_{d}\in Y_{n'}^{+}}\bigl(\widetilde{ \mathbf{M}}_{\alpha}(\vec {f}) \bigl(n',n_{d}+1 \bigr)-\widetilde{\mathbf{M}}_{\alpha}(\vec{f}) \bigl(n',n_{d} \bigr)\bigr) \\ &\quad{}+\sum_{n'\in\mathbb{Z}^{d-1}}\sum _{n_{d}\in Y_{n'}^{-}}\bigl(\widetilde{\mathbf{M}}_{\alpha}(\vec {f}) \bigl(n',n_{d}\bigr)-\widetilde{\mathbf{M}}_{\alpha}( \vec{f}) \bigl(n',n_{d}+1\bigr)\bigr) \\ &\lesssim_{\alpha,m,d}\prod_{i=1}^{m} \Vert f_{i} \Vert _{\ell^{1}(\mathbb{Z}^{d})}. \end{aligned} $$

This proves (3.8) and completes the proof of the boundedness part.

### Proof of Theorem 1.1—continuity part

#### Centered case

Let $\vec{f}=(f_{1},\ldots,f_{m})$ with each $f_{j}\in\ell^{1}(\mathbb{Z}^{d})$ and $g_{i,j}\rightarrow f_{j}$ in $\ell^{1}(\mathbb{Z}^{d})$ for any $1\leq j\leq m$ as $i\rightarrow\infty$. Let $\vec{g_{i}}=(g_{i,1},\ldots,g_{i,m})$ for $i\in\mathbb{Z}$. We may assume without loss of generality that all $g_{i,j}\geq0$ and $f_{j}\geq0$ since $||g_{i,j}|-|f_{j}| |\leq |g_{i,j}-f_{j} |$ for all $1\leq j\leq d$. Without loss of generality, we shall prove that

3.14 $$ \lim_{i\rightarrow\infty} \Vert D_{d}\mathbf{M}_{\alpha}( \vec{g_{i}}) -D_{d}\mathbf{M}_{\alpha}(\vec{f}) \Vert_{\ell^{1}(\mathbb {Z}^{d})}=0. $$

Given $\epsilon\in(0,1)$, there exists $N_{1}=N_{1}(\epsilon, \vec{f})\in\mathbb{N}$ such that

3.15 $$ \Vert g_{i,j}-f_{j} \Vert _{\ell^{1}(\mathbb{Z}^{d})}< \epsilon \quad\mbox{and}\quad \Vert g_{i,j} \Vert _{\ell^{1}(\mathbb{Z}^{d})} \leq \Vert f_{j} \Vert _{\ell^{1}(\mathbb{Z}^{d})}+1\quad \forall i\geq N_{1} \mbox{ and } 1\leq j\leq m. $$

By the boundedness part, we have that $D_{d}\mathbf{M}_{\alpha}(\vec{f})\in\ell^{1}(\mathbb{Z}^{d})$. We also note that $\alpha<{(m-1)d+1}$. Then, for above $\epsilon>0$, there exists an integer Λ with $\Lambda>\Lambda_{0}$ such that

3.16 $$ \max\Bigl\{ \bigl\Vert D_{d}\mathbf{M}_{\alpha}(\vec{f}) \chi_{\{ \Vert \vec{n} \Vert_{1}\geq4\Lambda\}} \bigr\Vert _{\ell^{1}(\mathbb{Z}^{d})}, \sup_{1\leq i\leq m} \Vert f_{i}\chi_{\{ \Vert\vec{n} \Vert _{1}\geq\Lambda\}} \Vert _{\ell^{1}(\mathbb{Z}^{d})},\Lambda ^{\alpha-(m-1)d-1}\Bigr\} < \epsilon. $$

One can easily check that

$$\begin{aligned}[b] &\bigl\vert \mathbf{M}_{\alpha}( \vec{g_{i}}) (\vec{n})-\mathbf {M}_{\alpha}(\vec{f}) (\vec{n}) \bigr\vert \\ &\quad \leq\sup_{r>0}N\bigl(\Gamma_{r}( \vec{n})\bigr)^{\frac{\alpha}{d}-m} \Biggl\vert \prod_{j=1}^{m} \sum_{\vec{k}\in\Gamma_{r}(\vec {n})}g_{i,j}(\vec{k})-\prod _{j=1}^{m}\sum_{\vec{k}\in\Gamma_{r}(\vec {n})}f_{j}( \vec{k}) \Biggr\vert \\ &\quad \leq\sum_{l=1}^{m}\Biggl(\prod _{\mu=1}^{l-1} \Vert f_{\mu} \Vert _{\ell^{1}(\mathbb{Z}^{d})}\Biggr) \Biggl(\prod_{\nu=l+1}^{m} \Vert g_{i,\nu} \Vert _{\ell ^{1}(\mathbb{Z}^{d})}\Biggr) \Vert g_{i,l}-f_{l} \Vert _{\ell ^{1}(\mathbb{Z}^{d})}\quad\forall\vec{n}\in \mathbb{Z}^{d}. \end{aligned} $$

This together with (3.15) implies that $\mathbf{M}_{\alpha}(\vec{g_{i}})(\vec{n})\rightarrow\mathbf{M}_{\alpha}(\vec{f}) (\vec{n})$ as $i\rightarrow\infty$ for any $\vec{n}\in \mathbb{Z}$. Therefore, we have

$$D_{d}\mathbf{M}_{\alpha}(\vec{g_{i}}) (\vec{n}) \rightarrow D_{d}\mathbf {M}_{\alpha}(\vec{f}) (\vec{n})\quad \mbox{as } i\rightarrow\infty \ \forall\vec{n}\in\mathbb{Z}^{d}. $$

It follows that there exists $N_{2}=N_{2}(\epsilon,\vec{f}, \Lambda)>0$ such that

3.17 $$ \bigl\vert D_{d}\mathbf{M}_{\alpha}(\vec{g_{i}}) ( \vec{n})-D_{d}\mathbf {M}_{\alpha}(\vec{f}) (\vec{n}) \bigr\vert \leq\frac{\epsilon }{N(\Gamma_{4\Lambda}(\vec{0}))}\quad\forall i\geq N_{2} \mbox{ and } \vec{n}\in\Gamma_{4\Lambda}(\vec{0}). $$

(3.17) together with (3.16) implies that

3.18 $$ \begin{aligned}[b] & \bigl\Vert D_{d} \mathbf{M}_{\alpha}(\vec{g_{i}})-D_{d} \mathbf{M}_{\alpha}(\vec{f}) \bigr\Vert _{\ell^{1}(\mathbb{Z}^{d})} \\ &\quad= \bigl\Vert \bigl(D_{d}\mathbf{M}_{\alpha}( \vec{g_{i}})-D_{d}\mathbf {M}_{\alpha}(\vec{f})\bigr) \chi_{\{ \Vert\vec{n} \Vert_{1}< 4\Lambda\}} \bigr\Vert _{\ell^{1}(\mathbb{Z}^{d})}\\ &\qquad {} + \bigl\Vert \bigl(D_{d}\mathbf{M}_{\alpha}(\vec{g_{i}})-D_{d} \mathbf{M}_{\alpha}(\vec{f})\bigr)\chi_{\{ \Vert\vec{n} \Vert_{1}\geq4\Lambda\}} \bigr\Vert _{\ell^{1}(\mathbb{Z}^{d})} \\ &\quad\leq2\epsilon+ \bigl\Vert D_{d}\mathbf{M}_{\alpha}(\vec {g_{i}})\chi_{\{ \Vert\vec{n} \Vert_{1}\geq4\Lambda\}} \bigr\Vert _{\ell^{1}(\mathbb{Z}^{d})}\quad\forall i\geq N_{2}. \end{aligned} $$

We now prove

3.19 $$ \bigl\Vert D_{d}\mathbf{M}_{\alpha}(\vec{g_{i}}) \chi_{\{ \Vert\vec{n} \Vert_{1}\geq2\Lambda\}} \bigr\Vert _{\ell^{1}(\mathbb {Z}^{d})}\lesssim_{\alpha,m,d,\vec{f}}\epsilon \quad\forall i\geq N_{1}. $$

Fix $i\geq N_{1}$. We can write

3.20 $$ \begin{aligned}[b]& \bigl\Vert D_{d}\mathbf{M}_{\alpha}( \vec{g_{i}})\chi_{\{ \Vert\vec{n} \Vert_{1}\geq4\Lambda\}} \bigr\Vert _{\ell^{1}(\mathbb{Z}^{d})}\\ &\quad \leq \sum _{ \Vert n' \Vert _{1}\geq2\Lambda\atop n'\in\mathbb {Z}^{d-1}}\sum_{n_{d}\in\mathbb{Z}} \bigl\vert \mathbf{M}_{\alpha}(\vec{g_{i}}) \bigl(n',n_{d}+1 \bigr)-\mathbf {M}_{\alpha}(\vec{g_{i}}) \bigl(n',n_{d} \bigr) \bigr\vert \\ &\qquad{}+\sum_{n'\in\mathbb{Z}^{d-1}}\sum _{ \vert n_{d} \vert \geq2\Lambda\atop n_{d}\in\mathbb{Z}} \bigl\vert \mathbf{M}_{\alpha}( \vec{g_{i}}) \bigl(n',n_{d}+1\bigr)-\mathbf {M}_{\alpha}(\vec{g_{i}}) \bigl(n',n_{d} \bigr) \bigr\vert \\ &\quad =:A_{1}+A_{2}. \end{aligned} $$

For $A_{1}$, fix $i\in\{1,2,\ldots,m\}$ and $n'\in\mathbb{Z}^{d-1}$ with $|n'|\geq2\Lambda$, let

$$\begin{aligned}& Z_{n'}=\bigl\{ n_{d}\in\mathbb{Z};\mathbf{M}_{\alpha}( \vec {g_{i}}) \bigl(n',n_{d}+1\bigr)= \mathbf{M}_{\alpha}(\vec{g_{i}}) \bigl(n',n_{d} \bigr)\bigr\} ; \\& Z_{n'}^{+}=\bigl\{ n_{d}\in\mathbb{Z}; \mathbf{M}_{\alpha}(\vec {g_{i}}) \bigl(n',n_{d}+1 \bigr)>\mathbf{M}_{\alpha}(\vec{g_{i}}) \bigl(n',n_{d} \bigr)\bigr\} ; \\& Z_{n'}^{-}=\bigl\{ n_{d}\in\mathbb{Z}; \mathbf{M}_{\alpha}(\vec {g_{i}}) \bigl(n',n_{d}+1 \bigr)< \mathbf{M}_{\alpha}(\vec{g_{i}}) \bigl(n',n_{d} \bigr)\bigr\} . \end{aligned}$$

We can write

3.21 $$ \begin{aligned}[b] A_{1}&=\sum _{ \Vert n' \Vert _{1}\geq2\Lambda\atop n'\in \mathbb{Z}^{d-1}}\sum_{n_{d}\in Z_{n'}^{+}}\bigl( \mathbf{M}_{\alpha}(\vec {g_{i}}) \bigl(n',n_{d}+1 \bigr)-\mathbf{M}_{\alpha}(\vec{g_{i}}) \bigl(n',n_{d} \bigr)\bigr) \\ &\quad{}+\sum_{ \Vert n' \Vert _{1}\geq2\Lambda\atop n'\in\mathbb{Z}^{d-1}}\sum _{n_{d}\in Z_{n'}^{-}}\bigl(\mathbf{M}_{\alpha}(\vec{g_{i}}) \bigl(n',n_{d}\bigr)-\mathbf{M}_{\alpha}( \vec{g_{i}}) \bigl(n',n_{d}+1\bigr)\bigr). \end{aligned} $$

By the arguments similar to those used in deriving (3.6), we have

3.22 $$ \begin{aligned}[b] &\sum_{ \Vert n' \Vert _{1}\geq2\Lambda\atop n'\in \mathbb{Z}^{d-1}}\sum _{n_{d}\in Z_{n'}^{+}}\bigl(\mathbf{M}_{\alpha}(\vec {g_{i}}) \bigl(n',n_{d}+1\bigr)- \mathbf{M}_{\alpha}(\vec{g_{i}}) \bigl(n',n_{d} \bigr)\bigr) \\ &\quad\leq\Biggl(\prod_{i=1}^{m-1} \Vert g_{i,j} \Vert _{\ell ^{1}(\mathbb{Z}^{d})}\Biggr)\sum _{ \Vert n' \Vert _{1}\geq2\Lambda \atop n'\in\mathbb{Z}^{d-1}}\sum_{n_{d}\in Z_{n'}^{+}}\sum _{\vec {k}\in\Gamma_{r(n',n_{d}+1)}(n',n_{d}+1)}\Phi\bigl(\bigl[r\bigl(n',n_{d}+1 \bigr)\bigr]\bigr)g_{i,m}(\vec {k}) \\ &\quad\leq\Biggl(\prod_{j=1}^{m-1} \Vert g_{i,j} \Vert _{\ell ^{1}(\mathbb{Z}^{d})}\Biggr)\sum _{\vec{k}\in\mathbb{Z}^{d}}g_{i,m}(\vec {k})I(\vec{k}), \end{aligned} $$

where

$$I(\vec{k}):=\sum_{ \Vert n' \Vert _{1}\geq2\Lambda\atop n'\in\mathbb{Z}^{d-1}}\sum _{n_{d}\in Z_{n'}^{+}}\Phi_{m-\frac{\alpha }{d}}\bigl(\bigl[r\bigl(n',n_{d}+1 \bigr)\bigr]\bigr)\chi_{\{ \Vert \vec{k}-(n',n_{d}+1) \Vert _{1}\leq[r(n',n_{d}+1)]\}}\bigl(n',n_{d}\bigr). $$

Fix $\vec{k}=(k',k_{d})\in\mathbb{Z}^{d}$. By a similar argument as that in getting (3.7), we can get

3.23 $$ I\bigl(k',k_{d}\bigr)\lesssim_{\alpha,m,d}1. $$

When $\Vert k' \Vert_{1}<\Lambda$ and $\Vert n' \Vert_{1}\geq2\Lambda$, then $\Vert\vec{k}-(n',n_{d}+1) \Vert_{1}\geq\Vert k'-n' \Vert\geq \Lambda$. Note that $m+\frac{1-\alpha}{d}>1$. Then, by Lemmas 2.1–2.2 and (3.16),

3.24 $$ \begin{aligned}[b] I\bigl(k',k_{d}\bigr)&\leq \sum_{ \Vert n' \Vert _{1}\geq2\Lambda \atop n'\in\mathbb{Z}^{d-1}}\sum_{n_{d}\in Z_{n'}^{+}} \Phi_{m-\frac {\alpha}{d}}\bigl(\bigl[r\bigl(n',n_{d}+1\bigr) \bigr]\bigr)\chi_{\{\Lambda\leq \Vert \vec {k}-(n',n_{d}+1) \Vert _{1}\leq[r(n',n_{d}+1)]\}}\bigl(n',n_{d}\bigr)\hspace{-20pt} \\ &\leq\sum_{ \Vert n' \Vert _{1}\geq2\Lambda\atop n'\in \mathbb{Z}^{d-1}}\sum_{n_{d}\in Z_{n'}^{+}} \Phi_{m-\frac{\alpha }{d}}\bigl( \bigl\Vert \vec{k}-\bigl(n',n_{d}+1 \bigr) \bigr\Vert _{1}\bigr)\chi_{\{\Lambda \leq \Vert \vec{k}-(n',n_{d}+1) \Vert _{1}\}}\bigl(n',n_{d} \bigr) \\ &\leq\sum_{ \Vert \vec{n} \Vert _{1}\geq\Lambda}\Phi _{m-\frac{\alpha}{d}}\bigl( \Vert \vec{n} \Vert _{1}\bigr) \lesssim_{\alpha,m,d}\sum _{ \Vert \vec{n} \Vert _{1}\geq \Lambda}\bigl(N_{1,d}\bigl( \Vert \vec{n} \Vert _{1}\bigr)\bigr)^{\frac {\alpha-1}{d}-m} \\ &\lesssim_{\alpha,m,d}\Lambda^{\alpha-(m-1)d-1}\lesssim_{\alpha ,m,d}\epsilon. \end{aligned} $$

Combining (3.24) with (3.23) and (3.15)–(3.16) implies that

$$\begin{aligned} &\sum_{\vec{k}\in\mathbb{Z}^{d}}g_{i,m}(\vec{k})I(\vec{k}) \\ &\quad \leq\sum_{ \Vert k' \Vert _{1}\geq\Lambda}\sum_{k_{d}\in \mathbb{Z}^{d}}g_{i,m} \bigl(k',k_{d}\bigr)I\bigl(k',k_{d} \bigr)+ \sum_{ \Vert k' \Vert _{1}< \Lambda}\sum_{k_{d}\in\mathbb {Z}^{d}}g_{i,m} \bigl(k',k_{d}\bigr)I\bigl(k',k_{d} \bigr) \\ &\quad \lesssim_{\alpha,m,d}\bigl( \Vert g_{i,m}\chi_{\{ \Vert\vec{k} \Vert_{1}\geq\Lambda\}} \Vert _{\ell^{1}(\mathbb{Z}^{d})}+ \Vert g_{i,m} \Vert _{\ell^{1}(\mathbb{Z}^{d})}\epsilon \bigr) \\ &\quad \lesssim_{\alpha,m,d}\bigl( \bigl\Vert (g_{i,m}-f_{m}) \chi_{\{ \Vert\vec {k} \Vert_{1}\geq\Lambda\}} \bigr\Vert _{\ell^{1}(\mathbb{Z}^{d})}+ \Vert f_{m} \chi_{\{ \Vert\vec{k} \Vert_{1}\geq\Lambda\}} \Vert _{\ell^{1}(\mathbb{Z}^{d})}+\bigl( \Vert f_{m} \Vert _{\ell ^{1}(\mathbb{Z}^{d})}+1\bigr)\epsilon\bigr) \\ &\quad \lesssim_{\alpha,m,d,f_{m}} \epsilon. \end{aligned}$$

This together with (3.22) and (3.15) yields that

3.25 $$ \sum_{ \Vert n' \Vert _{1}\geq2\Lambda\atop n'\in\mathbb {Z}^{d-1}}\sum_{n_{d}\in Z_{n'}^{+}} \bigl(\mathbf{M}_{\alpha}(\vec {g_{i}}) \bigl(n',n_{d}+1 \bigr)-\mathbf{M}_{\alpha}(\vec{g_{i}}) \bigl(n',n_{d} \bigr)\bigr)\lesssim _{\alpha,m,d,\vec{f}}\epsilon. $$

Similarly,

3.26 $$ \sum_{ \Vert n' \Vert _{1}\geq2\Lambda\atop n'\in\mathbb {Z}^{d-1}}\sum_{n_{d}\in Z_{n'}^{-}} \bigl(\mathbf{M}_{\alpha}(\vec {g_{i}}) \bigl(n',n_{d} \bigr)-\mathbf{M}_{\alpha}(\vec{g_{i}}) \bigl(n',n_{d}+1 \bigr)\bigr)\lesssim _{\alpha,m,d,\vec{f}}\epsilon. $$

It follows from (3.21) and (3.25)–(3.26) that

3.27 $$ A_{1}\lesssim_{\alpha,m,d,\vec{f}}\epsilon\quad\forall i\geq N_{1}. $$

By the arguments similar to those used to derive (3.27),

3.28 $$ A_{2}\lesssim_{\alpha,m,d,\vec{f}}\epsilon\quad\forall i\geq N_{1}. $$

Then (3.19) follows from (3.20) and (3.27)–(3.28). From (3.18) and (3.19) we have

$$\bigl\Vert D_{d}\mathbf{M}_{\alpha}(\vec{g_{i}})-D_{d} \mathbf{M}_{\alpha}(\vec{f}) \bigr\Vert _{\ell^{1}(\mathbb{Z}^{d})} \lesssim_{\alpha ,m,d,\vec{f}}\epsilon\quad\forall i\geq\max\{N_{1},N_{2} \}, $$

which yields (3.14).

#### Uncentered case

The proof is essentially analogous to Section 3.2.1. We leave the details to the interested reader.
